# 6-Fluoro-4-oxo-4*H*-chromene-3-carbalde­hyde

**DOI:** 10.1107/S1600536814008502

**Published:** 2014-04-18

**Authors:** Yoshinobu Ishikawa

**Affiliations:** a School of Pharmaceutical Sciences, University of Shizuoka, 52-1 Yada, Suruga-ku, Shizuoka 422-8526, Japan

## Abstract

In the title compound, C_10_H_5_FO_3_, the non-H atoms are essentially coplanar (r.m.s. deviation = 0.0071 Å), with the largest deviation from the mean plane [0.0203 (15) Å] being found for the ring C atom in the 2-position. In the crystal, mol­ecules are linked into a three-dimensional architecture *via* C—H⋯O hydrogen bonds and π–π stacking inter­actions between the chromone units along the *a*-axis direction [centroid–centroid distance between the benzene and pyran rings = 3.707 (2) Å].

## Related literature   

For related structures, see: Ishikawa (2014*a* ▶,*b* ▶). For halogen bonding, see: Auffinger *et al.* (2004 ▶); Metrangolo *et al.* (2005 ▶); Wilcken *et al.* (2013 ▶); Sirimulla *et al.* (2013 ▶).
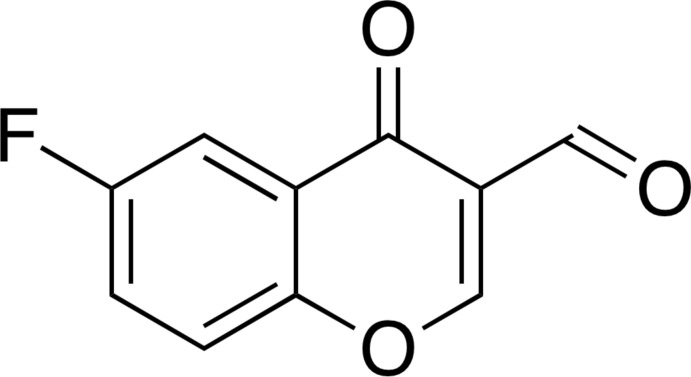


## Experimental   

### 

#### Crystal data  

C_10_H_5_FO_3_
*M* *_r_* = 192.15
Monoclinic, 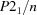

*a* = 7.8530 (19) Å
*b* = 5.6020 (17) Å
*c* = 17.987 (5) Å
β = 95.09 (2)°
*V* = 788.2 (4) Å^3^
*Z* = 4
Mo *K*α radiation
μ = 0.14 mm^−1^
*T* = 100 K
0.30 × 0.20 × 0.12 mm

#### Data collection  

Rigaku AFC-7R diffractometer
2412 measured reflections
1815 independent reflections
1496 reflections with *F* ^2^ > 2σ(*F* ^2^)
*R*_int_ = 0.024
3 standard reflections every 150 reflections intensity decay: 3.4%

#### Refinement  

*R*[*F* ^2^ > 2σ(*F* ^2^)] = 0.036
*wR*(*F* ^2^) = 0.106
*S* = 1.03
1815 reflections
128 parameters
H-atom parameters constrained
Δρ_max_ = 0.37 e Å^−3^
Δρ_min_ = −0.22 e Å^−3^

### 

Data collection: *WinAFC Diffractometer Control Software* (Rigaku, 1999 ▶); cell refinement: *WinAFC Diffractometer Control Software*; data reduction: *WinAFC Diffractometer Control Software*; program(s) used to solve structure: *SIR2008* (Burla *et al.*, 2007 ▶); program(s) used to refine structure: *SHELXL97* (Sheldrick, 2008 ▶); molecular graphics: *CrystalStructure* (Rigaku, 2010 ▶); software used to prepare material for publication: *CrystalStructure*.

## Supplementary Material

Crystal structure: contains datablock(s) General, I. DOI: 10.1107/S1600536814008502/tk5307sup1.cif

Structure factors: contains datablock(s) I. DOI: 10.1107/S1600536814008502/tk5307Isup2.hkl

Supporting information file. DOI: 10.1107/S1600536814008502/tk5307Isup3.cml

CCDC reference: 997449

Additional supporting information:  crystallographic information; 3D view; checkCIF report

## Figures and Tables

**Table 1:** Hydrogen-bond geometry (Å, °)

*D*—H⋯*A*	*D*—H	H⋯*A*	*D*⋯*A*	*D*—H⋯*A*
C1—H1⋯O3^i^	0.95	2.37	3.308 (2)	171
C4—H2⋯O2^ii^	0.95	2.35	3.235 (2)	154
C6—H3⋯O3^iii^	0.95	2.48	3.198 (2)	133

Symmetry codes: (i) 
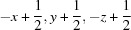
; (ii) 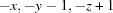; (iii) 
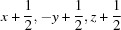
.

**Fig. 1.**
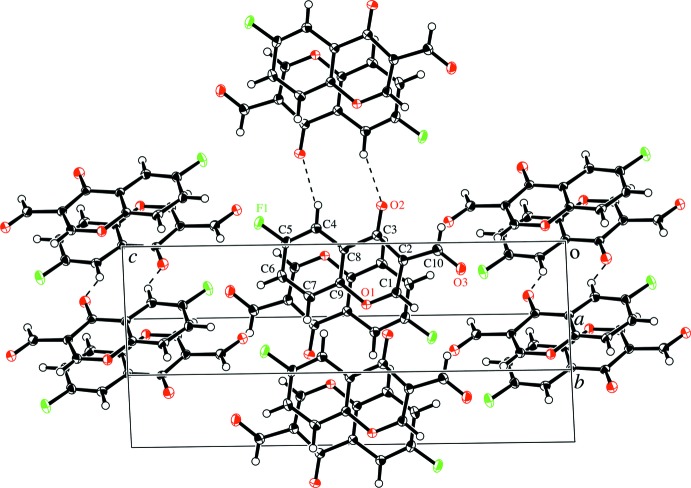
A packing view of the title compound with displacement ellipsoids drawn at the 50% probability level. Hydrogen atoms are shown as small spheres of arbitrary radius. The shortest intermolecular C—H···O hydrogen bonds are represented as dashed lines.

**Fig. 2.**
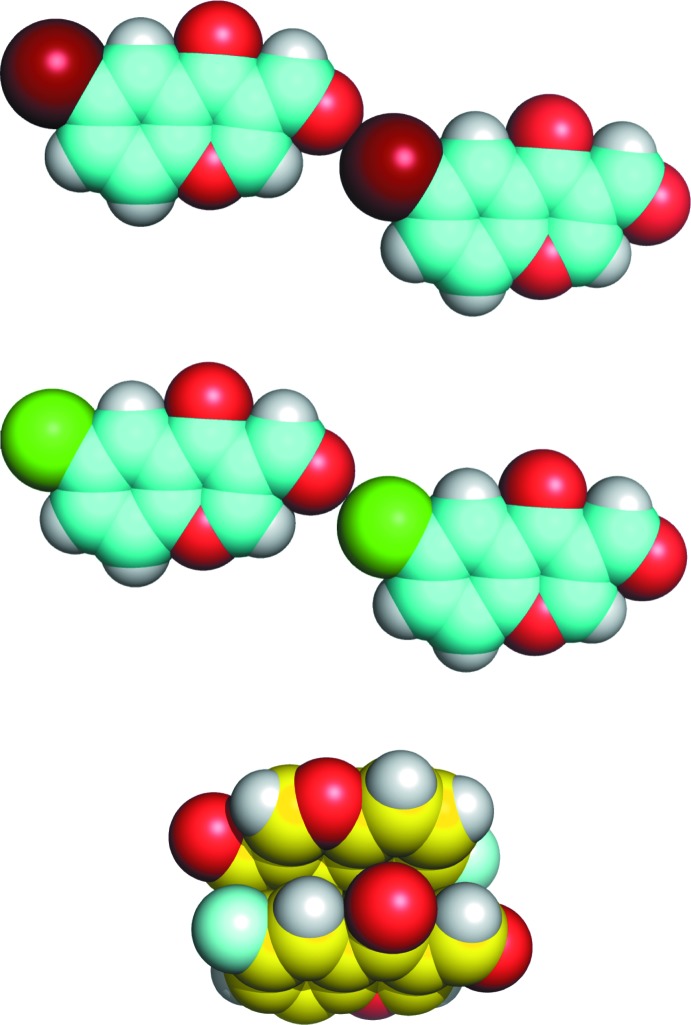
Sphere models of the crystal structures of 6-bromo-4-oxo-4H-chromene-3-carbaldehyde (top), 6-chloro-4-oxo-4H-chromene-3-carbaldehyde (middle), and the title compound (bottom).

## supplementary crystallographic information

### 1. Structural commentary

Halogen bonds have been found to occur in organic, inorganic and biological systems, and have recently attracted much attention in medicinal chemistry, chemical biology and supra­molecular chemistry (Auffinger *et al.*, 2004, Metrangolo *et al.*, 2005, Wilcken *et al.*, 2013, Sirimulla *et al.*, 2013). We have recently reported the crystal structures of monohalogenated 3-formyl­chromone derivatives 6-bromo-4-oxo-4*H*-chromene-3-carbaldehyde (Ishikawa, 2014*a*) and 6-chloro-4-oxo-4*H*-chromene-3-carbaldehyde (Ishikawa, 2014*b*). Halogen bonding is observed in the former, but is not observed in the latter. As part of our inter­est in this type of chemical bonding, we herein report the crystal structure of a monofluorinated 3-formyl­chromone derivative, 6-fluoro-4-oxo-4*H*-chromene-3-carbaldehyde. The objective of this study is to reveal whether halogen bond(*s*) can be formed in the crystal structure of this compound with a fluorine atom at the 6-position.

The mean deviation of the least-square planes for the non-hydrogen atoms is 0.0071 Å, and the largest deviation is 0.0203 (15) Å for C1 (Fig. 1). This mean that the atoms are essentially coplanar. In the crystal, the molecules are linked *via* C—H···O hydrogen bonds and are further linked by π—π stacking inter­actions between the chromone units [centroid–centroid distance between the benzene and pyran rings = 3.707 (2) Å], into a three-dimensional architectureFig. 1. The distance between the fluorine and proximal formyl oxygen atoms [3.498 (2) Å, Fig.2 (bottom)] is far from halogen bonding. The fact that halogen bonding is observed in the crystal structure of 6-bromo-4-oxo-4*H*-chromene-3-carbaldehyde (Fig. 2 (top)) but is not observed in those of 6-chloro-4-oxo-4*H*-chromene-3-carbaldehyde (Fig. 2 (middle)) and the title compound demonstrates the trend of the size of σ-hole on halogens (Br > Cl > F; Sirimulla *et al.*, 2013).

### 2. Synthesis and crystallization

Single crystals suitable for X-ray diffraction were obtained by slow evaporation at room temperature of an ethyl acetate solution of the commercially available title compound.

### 3. Refinement

The C(*sp*^2^)-bound hydrogen atoms were placed in geometrical positions [C—H = 0.95 Å, *U*_iso_(H) = 1.2*U*_eq_(C)], and refined using a riding model. One reflection (0 1 1) was omitted because of systematic error.

### Crystal data

**Table:**

C_10_H_5_FO_3_	*F*(000) = 392.00
*M**_r_* = 192.15	*D*_x_ = 1.619 Mg m^−^^3^
Monoclinic, *P*2_1_/*n*	Mo *K*α radiation, λ = 0.71069 Å
Hall symbol: -P 2yn	Cell parameters from 25 reflections
*a* = 7.8530 (19) Å	θ = 15.2–17.3°
*b* = 5.6020 (17) Å	µ = 0.14 mm^−^^1^
*c* = 17.987 (5) Å	*T* = 100 K
β = 95.09 (2)°	Plate, colourless
*V* = 788.2 (4) Å^3^	0.30 × 0.20 × 0.12 mm
*Z* = 4	

### Data collection

**Table:**

Rigaku AFC-7R diffractometer	θ_max_ = 27.5°
ω–2θ scans	*h* = −10→10
2412 measured reflections	*k* = −4→7
1815 independent reflections	*l* = −23→13
1496 reflections with *F*^2^ > 2σ(*F*^2^)	3 standard reflections every 150 reflections
*R*_int_ = 0.024	intensity decay: 3.4%

### Refinement

**Table:**

Refinement on *F*^2^	Hydrogen site location: inferred from neighbouring sites
*R*[*F*^2^ > 2σ(*F*^2^)] = 0.036	H-atom parameters constrained
*wR*(*F*^2^) = 0.106	*w* = 1/[σ^2^(*F*_o_^2^) + (0.0562*P*)^2^ + 0.3686*P*] where *P* = (*F*_o_^2^ + 2*F*_c_^2^)/3
*S* = 1.03	(Δ/σ)_max_ < 0.001
1815 reflections	Δρ_max_ = 0.37 e Å^−^^3^
128 parameters	Δρ_min_ = −0.22 e Å^−^^3^
0 restraints	Extinction correction: *SHELXL97* (Sheldrick, 2008)
Primary atom site location: structure-invariant direct methods	Extinction coefficient: 0.011 (3)
Secondary atom site location: difference Fourier map	

### Special details

**Table:**

Refinement. Refinement was performed using all reflections. The weighted *R*-factor (*wR*) and goodness of fit (*S*) are based on *F*^2^. *R*-factor (gt) are based on *F*. The threshold expression of *F*^2^ > 2.0 σ(*F*^2^) is used only for calculating *R*-factor (gt).

### Fractional atomic coordinates and isotropic or equivalent isotropic displacement parameters (Å^2^)

**Table:**

	*x*	*y*	*z*	*U*_iso_*/*U*_eq_	
F1	0.24629 (11)	−0.31171 (17)	0.68919 (5)	0.0261 (3)	
O1	0.33585 (12)	0.28450 (17)	0.45542 (5)	0.0178 (3)	
O2	0.05794 (13)	−0.32557 (18)	0.41030 (5)	0.0205 (3)	
O3	0.13190 (13)	0.13025 (19)	0.23839 (5)	0.0226 (3)	
C1	0.26630 (17)	0.2332 (3)	0.38638 (8)	0.0169 (3)	
C2	0.17422 (16)	0.0346 (3)	0.36799 (7)	0.0155 (3)	
C3	0.14094 (16)	−0.1427 (3)	0.42424 (7)	0.0147 (3)	
C4	0.19425 (16)	−0.2340 (3)	0.56069 (7)	0.0163 (3)	
C5	0.26679 (17)	−0.1674 (3)	0.62980 (7)	0.0185 (3)	
C6	0.36078 (17)	0.0417 (3)	0.64257 (8)	0.0191 (3)	
C7	0.38313 (17)	0.1905 (3)	0.58328 (8)	0.0182 (3)	
C8	0.21614 (16)	−0.0827 (3)	0.50018 (7)	0.0142 (3)	
C9	0.30989 (16)	0.1270 (3)	0.51258 (7)	0.0147 (3)	
C10	0.10652 (17)	−0.0034 (3)	0.28948 (7)	0.0187 (3)	
H1	0.2830	0.3442	0.3478	0.0203*	
H2	0.1311	−0.3782	0.5541	0.0195*	
H3	0.4087	0.0810	0.6914	0.0229*	
H4	0.4472	0.3338	0.5904	0.0218*	
H5	0.0388	−0.1414	0.2783	0.0224*	

### Atomic displacement parameters (Å^2^)

**Table:**

	*U*^11^	*U*^22^	*U*^33^	*U*^12^	*U*^13^	*U*^23^
F1	0.0315 (5)	0.0312 (5)	0.0153 (5)	−0.0033 (4)	0.0012 (4)	0.0069 (4)
O1	0.0222 (5)	0.0154 (5)	0.0157 (5)	−0.0035 (4)	0.0014 (4)	0.0000 (4)
O2	0.0223 (5)	0.0183 (5)	0.0204 (5)	−0.0056 (4)	−0.0003 (4)	−0.0014 (4)
O3	0.0293 (6)	0.0222 (6)	0.0161 (5)	0.0045 (5)	0.0013 (4)	0.0016 (4)
C1	0.0186 (7)	0.0171 (7)	0.0153 (6)	0.0018 (5)	0.0033 (5)	0.0010 (5)
C2	0.0146 (6)	0.0166 (7)	0.0154 (7)	0.0024 (5)	0.0026 (5)	−0.0014 (5)
C3	0.0125 (6)	0.0151 (6)	0.0167 (7)	0.0024 (5)	0.0021 (5)	−0.0014 (5)
C4	0.0149 (6)	0.0163 (7)	0.0178 (7)	0.0001 (5)	0.0029 (5)	−0.0001 (6)
C5	0.0192 (7)	0.0215 (7)	0.0152 (7)	0.0032 (6)	0.0042 (5)	0.0033 (6)
C6	0.0177 (6)	0.0246 (8)	0.0147 (6)	0.0027 (6)	−0.0003 (5)	−0.0034 (6)
C7	0.0163 (7)	0.0177 (7)	0.0204 (7)	0.0002 (5)	0.0010 (5)	−0.0051 (6)
C8	0.0120 (6)	0.0154 (6)	0.0154 (7)	0.0019 (5)	0.0023 (5)	−0.0015 (5)
C9	0.0146 (6)	0.0151 (7)	0.0149 (6)	0.0016 (5)	0.0032 (5)	0.0002 (5)
C10	0.0190 (7)	0.0197 (7)	0.0171 (7)	0.0024 (6)	0.0005 (5)	−0.0015 (6)

### Geometric parameters (Å, º)

**Table:**

F1—C5	1.3606 (17)	C4—C8	1.4020 (19)
O1—C1	1.3427 (17)	C5—C6	1.393 (2)
O1—C9	1.3835 (17)	C6—C7	1.377 (2)
O2—C3	1.2282 (17)	C7—C9	1.3946 (19)
O3—C10	1.2155 (17)	C8—C9	1.394 (2)
C1—C2	1.352 (2)	C1—H1	0.950
C2—C3	1.4579 (19)	C4—H2	0.950
C2—C10	1.4796 (18)	C6—H3	0.950
C3—C8	1.4780 (18)	C7—H4	0.950
C4—C5	1.3723 (18)	C10—H5	0.950
			
O1···C3	2.8691 (18)	C4···H3	3.2823
O2···C1	3.576 (2)	C5···H4	3.2513
O2···C4	2.8658 (17)	C6···H2	3.2881
O2···C10	2.8760 (18)	C8···H4	3.2937
O3···C1	2.8344 (19)	C9···H1	3.1941
C1···C7	3.587 (3)	C9···H2	3.2746
C1···C8	2.760 (2)	C9···H3	3.2509
C2···C9	2.7704 (19)	C10···H1	2.5600
C4···C7	2.813 (2)	H1···H5	3.4953
C5···C9	2.721 (2)	H3···H4	2.3436
C6···C8	2.796 (2)	F1···H3^i^	2.6307
F1···F1^i^	3.5513 (16)	F1···H4^vi^	3.1761
F1···F1^ii^	3.5513 (16)	F1···H5^iv^	3.4684
F1···O2^iii^	3.5044 (15)	F1···H5^v^	2.6941
F1···O3^iv^	3.4981 (16)	O1···H2^vii^	3.1318
F1···O3^v^	3.5584 (16)	O1···H4^xiv^	2.8987
F1···C6^i^	3.315 (2)	O2···H1^vi^	2.8570
F1···C7^vi^	3.5947 (19)	O2···H2^iii^	2.3533
F1···C10^iv^	3.3360 (18)	O3···H1^x^	2.3664
F1···C10^v^	3.3800 (18)	O3···H3^xii^	2.4781
O1···O2^vii^	3.1431 (15)	O3···H4^xii^	2.9229
O1···C3^vii^	3.5781 (19)	O3···H5^xi^	2.9244
O1···C4^vii^	3.5315 (19)	C1···H2^iv^	3.4844
O1···C6^viii^	3.5869 (19)	C1···H3^viii^	3.4931
O1···C7^viii^	3.5649 (19)	C1···H4^xiv^	3.3095
O2···F1^iii^	3.5044 (15)	C1···H5^xi^	3.5228
O2···O1^vi^	3.1431 (15)	C2···H2^iv^	3.4665
O2···C1^vi^	3.0162 (19)	C2···H3^viii^	3.5928
O2···C4^iii^	3.2351 (19)	C3···H1^vi^	3.4163
O2···C7^iv^	3.5572 (19)	C3···H2^iii^	3.4741
O2···C9^iv^	3.4951 (19)	C3···H4^viii^	3.4389
O3···F1^iv^	3.4981 (16)	C4···H4^vi^	3.1477
O3···F1^ix^	3.5584 (16)	C5···H4^vi^	3.2399
O3···O3^x^	3.3650 (17)	C5···H5^iv^	3.4912
O3···O3^xi^	3.3650 (17)	C5···H5^v^	3.4391
O3···C1^x^	3.3077 (19)	C6···H1^viii^	3.5277
O3···C2^xi^	3.4099 (18)	C6···H5^v^	3.5118
O3···C6^xii^	3.1985 (18)	C7···H2^vii^	3.1377
O3···C7^xii^	3.4120 (19)	C8···H4^viii^	3.5193
O3···C10^xi^	2.9773 (19)	C9···H2^vii^	3.2243
C1···O2^vii^	3.0162 (19)	C10···H1^x^	2.8205
C1···O3^xi^	3.3077 (19)	C10···H3^xii^	3.2620
C1···C6^viii^	3.390 (2)	H1···O2^vii^	2.8570
C2···O3^x^	3.4099 (18)	H1···O3^xi^	2.3664
C2···C4^iv^	3.453 (2)	H1···C3^vii^	3.4163
C2···C5^iv^	3.546 (2)	H1···C6^viii^	3.5277
C3···O1^vi^	3.5781 (19)	H1···C10^xi^	2.8205
C3···C4^iv^	3.403 (2)	H1···H3^viii^	3.5122
C3···C8^iv^	3.461 (2)	H1···H4^xiv^	2.9261
C4···O1^vi^	3.5315 (19)	H1···H5^xi^	2.7690
C4···O2^iii^	3.2351 (19)	H2···O1^vi^	3.1318
C4···C2^iv^	3.453 (2)	H2···O2^iii^	2.3533
C4···C3^iv^	3.403 (2)	H2···C1^iv^	3.4844
C4···C7^vi^	3.557 (3)	H2···C2^iv^	3.4665
C5···C2^iv^	3.546 (2)	H2···C3^iii^	3.4741
C5···C10^iv^	3.517 (2)	H2···C7^vi^	3.1377
C6···F1^ii^	3.315 (2)	H2···C9^vi^	3.2243
C6···O1^viii^	3.5869 (19)	H2···H2^iii^	3.0288
C6···O3^xiii^	3.1985 (18)	H2···H4^vi^	2.9843
C6···C1^viii^	3.390 (2)	H3···F1^ii^	2.6307
C7···F1^vii^	3.5947 (19)	H3···O3^xiii^	2.4781
C7···O1^viii^	3.5649 (19)	H3···C1^viii^	3.4931
C7···O2^iv^	3.5572 (19)	H3···C2^viii^	3.5928
C7···O3^xiii^	3.4120 (19)	H3···C10^xiii^	3.2620
C7···C4^vii^	3.557 (3)	H3···H1^viii^	3.5122
C7···C9^viii^	3.562 (2)	H3···H5^v^	3.0440
C8···C3^iv^	3.461 (2)	H4···F1^vii^	3.1761
C8···C8^iv^	3.518 (2)	H4···O1^xiv^	2.8987
C9···O2^iv^	3.4951 (19)	H4···O3^xiii^	2.9229
C9···C7^viii^	3.562 (2)	H4···C1^xiv^	3.3095
C9···C9^viii^	3.374 (2)	H4···C3^viii^	3.4389
C10···F1^iv^	3.3360 (18)	H4···C4^vii^	3.1477
C10···F1^ix^	3.3800 (18)	H4···C5^vii^	3.2399
C10···O3^x^	2.9773 (19)	H4···C8^viii^	3.5193
C10···C5^iv^	3.517 (2)	H4···H1^xiv^	2.9261
F1···H2	2.5447	H4···H2^vii^	2.9843
F1···H3	2.5415	H5···F1^iv^	3.4684
O1···H4	2.5219	H5···F1^ix^	2.6941
O2···H2	2.6151	H5···O3^x^	2.9244
O2···H5	2.5814	H5···C1^x^	3.5228
O3···H1	2.5100	H5···C5^iv^	3.4912
C1···H5	3.2808	H5···C5^ix^	3.4391
C3···H1	3.2935	H5···C6^ix^	3.5118
C3···H2	2.6889	H5···H1^x^	2.7690
C3···H5	2.6767	H5···H3^ix^	3.0440
			
C1—O1—C9	118.43 (11)	C4—C8—C9	118.99 (12)
O1—C1—C2	124.57 (13)	O1—C9—C7	116.03 (12)
C1—C2—C3	121.05 (12)	O1—C9—C8	121.94 (11)
C1—C2—C10	119.43 (13)	C7—C9—C8	122.02 (13)
C3—C2—C10	119.53 (12)	O3—C10—C2	124.30 (13)
O2—C3—C2	123.45 (12)	O1—C1—H1	117.713
O2—C3—C8	122.66 (12)	C2—C1—H1	117.717
C2—C3—C8	113.89 (12)	C5—C4—H2	121.008
C5—C4—C8	117.98 (13)	C8—C4—H2	121.007
F1—C5—C4	118.76 (13)	C5—C6—H3	120.543
F1—C5—C6	117.91 (12)	C7—C6—H3	120.538
C4—C5—C6	123.32 (13)	C6—C7—H4	120.618
C5—C6—C7	118.92 (13)	C9—C7—H4	120.626
C6—C7—C9	118.76 (13)	O3—C10—H5	117.845
C3—C8—C4	120.90 (12)	C2—C10—H5	117.850
C3—C8—C9	120.11 (12)		
			
C1—O1—C9—C7	−179.22 (10)	C8—C4—C5—F1	179.77 (11)
C1—O1—C9—C8	0.80 (17)	C8—C4—C5—C6	−0.1 (2)
C9—O1—C1—C2	−1.26 (19)	H2—C4—C5—F1	−0.2
C9—O1—C1—H1	178.7	H2—C4—C5—C6	179.9
O1—C1—C2—C3	0.9 (2)	H2—C4—C8—C3	0.3
O1—C1—C2—C10	−179.01 (11)	H2—C4—C8—C9	−179.9
H1—C1—C2—C3	−179.1	F1—C5—C6—C7	−179.98 (11)
H1—C1—C2—C10	1.0	F1—C5—C6—H3	0.0
C1—C2—C3—O2	179.92 (12)	C4—C5—C6—C7	−0.1 (2)
C1—C2—C3—C8	0.01 (18)	C4—C5—C6—H3	179.9
C1—C2—C10—O3	2.2 (2)	C5—C6—C7—C9	0.4 (2)
C1—C2—C10—H5	−177.8	C5—C6—C7—H4	−179.6
C3—C2—C10—O3	−177.66 (12)	H3—C6—C7—C9	−179.6
C3—C2—C10—H5	2.3	H3—C6—C7—H4	0.4
C10—C2—C3—O2	−0.21 (19)	C6—C7—C9—O1	179.57 (11)
C10—C2—C3—C8	179.87 (11)	C6—C7—C9—C8	−0.5 (2)
O2—C3—C8—C4	−0.5 (2)	H4—C7—C9—O1	−0.4
O2—C3—C8—C9	179.68 (12)	H4—C7—C9—C8	179.6
C2—C3—C8—C4	179.39 (11)	C3—C8—C9—O1	0.01 (19)
C2—C3—C8—C9	−0.40 (17)	C3—C8—C9—C7	−179.97 (11)
C5—C4—C8—C3	−179.74 (11)	C4—C8—C9—O1	−179.78 (11)
C5—C4—C8—C9	0.05 (19)	C4—C8—C9—C7	0.24 (19)

Symmetry codes: (i) −*x*+1/2, *y*−1/2, −*z*+3/2; (ii) −*x*+1/2, *y*+1/2, −*z*+3/2; (iii) −*x*, −*y*−1, −*z*+1; (iv) −*x*, −*y*, −*z*+1; (v) *x*+1/2, −*y*−1/2, *z*+1/2; (vi) *x*, *y*−1, *z*; (vii) *x*, *y*+1, *z*; (viii) −*x*+1, −*y*, −*z*+1; (ix) *x*−1/2, −*y*−1/2, *z*−1/2; (x) −*x*+1/2, *y*−1/2, −*z*+1/2; (xi) −*x*+1/2, *y*+1/2, −*z*+1/2; (xii) *x*−1/2, −*y*+1/2, *z*−1/2; (xiii) *x*+1/2, −*y*+1/2, *z*+1/2; (xiv) −*x*+1, −*y*+1, −*z*+1.

### Hydrogen-bond geometry (Å, º)

**Table:**

*D*—H···*A*	*D*—H	H···*A*	*D*···*A*	*D*—H···*A*
C1—H1···O3^xi^	0.95	2.37	3.308 (2)	171
C4—H2···O2^iii^	0.95	2.35	3.235 (2)	154
C6—H3···O3^xiii^	0.95	2.48	3.198 (2)	133

Symmetry codes: (iii) −*x*, −*y*−1, −*z*+1; (xi) −*x*+1/2, *y*+1/2, −*z*+1/2; (xiii) *x*+1/2, −*y*+1/2, *z*+1/2.
